# Disease implication of hyper-Hippo signalling

**DOI:** 10.1098/rsob.160119

**Published:** 2016-10-19

**Authors:** Shu-Ping Wang, Lan-Hsin Wang

**Affiliations:** 1 Laboratory of Biochemistry and Molecular Biology, The Rockefeller University, New York, NY 10065, USA; 2 Graduate Institute of Life Sciences, National Defense Medical Center, 161, Sec. 6, Minquan E. Rd., Neihu Dist, Taipei City 114, Taiwan

**Keywords:** Hippo pathway, arrhythmogenic cardiomyopathy, Sveinsson's chorioretinal atrophy, Alzheimer's disease, amyotrophic lateral sclerosis, diabetes

## Abstract

The Hippo signalling pathway regulates cellular proliferation, apoptosis and differentiation, thus exerting profound effects on cellular homeostasis. Inhibition of Hippo signalling has been frequently implicated in human cancers, indicating a well-known tumour suppressor function of the Hippo pathway. However, it is less certain whether and how hyperactivation of the Hippo pathway affects biological outcome in living cells. This review describes current knowledge of the regulatory mechanisms of the Hippo pathway, mainly focusing on hyperactivation of the Hippo signalling nexus. The disease implications of hyperactivated Hippo signalling have also been discussed, including arrhythmogenic cardiomyopathy, Sveinsson's chorioretinal atrophy, Alzheimer's disease, amyotrophic lateral sclerosis and diabetes. By highlighting the significance of disease-relevant Hippo signalling activation, this review can offer exciting prospects to address the onset and potential reversal of Hippo-related disorders.

## 1. Introduction: an overview of Hippo pathway regulation

**Figure 1.**
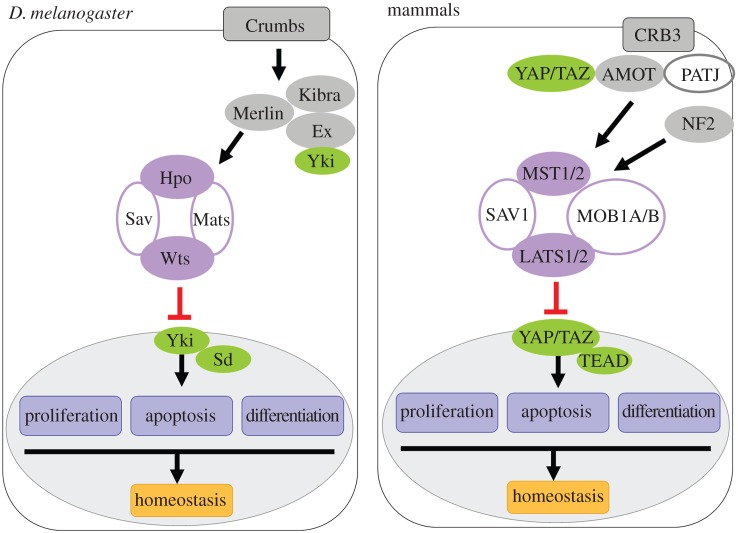
Models of Hippo pathway in fly and mammals. A simplified version of Hippo pathway regulation is shown here. In both *Drosophila* and mammals, when Yki/YAP/TAZ is relieved from inhibition through phosphorylation-dependent or independent mechanisms, its nuclear translocation then drives target gene expression in regulation of cellular proliferation, apoptosis and differentiation. The phosphorylation mechanism relies on the core kinase cascade including Hpo/MST, Wts/LATS, Sav/SAV1 and Mats/MOB1. In *Drosophila*, the FERM domain protein Ex has been shown to physically associate with Yki and block its nuclear translocation. Similarly, in mammals, the adherens protein AMOT and CRB3 complex inhibit target gene expression via sequestering YAP/TAZ in cytoplasm.

The tumour suppressor Hippo pathway has emerged as a major regulator of organ size control, stem cell pluripotency and regeneration (figure 1). Deregulation of Hippo signalling leads to deleterious consequences including cancers [1–6]. Deregulation of the Hippo pathway often refers to inhibition of Hippo signal transduction and over-proliferative effects. Nevertheless, the Hippo pathway can also be deregulated in an opposite way, which causes hyperactivation of Hippo signalling and survival defects (table 1). In contrast with the inactive Hippo pathway, the biological significance of hyperactive Hippo signalling has been largely underestimated.

**Table 1.** Hyperactive Hippo pathway and diseases.

disease	Hippo components	affected organ or tissue	evidence	references
arrhythmogenic cardiomyopathy	NF2, MST1/2, LATS1/2 and YAP	heart	gene expression	[7]
Sveinsson's chorioretinal atrophy	TEAD1	eye	human, mouse mutation	[8,9]
retinal detachment	MST2	eye	gene expression	[10]
Alzheimer's disease	MST1/2, YAP	brain, nerves	gene expression	[11,12]
skeletal muscle atrophy	MST1, YAP?	muscles, nerves	gene expression	[13]
amyotrophic lateral sclerosis	MST1, YAP	nerves	gene expression	[14,15]
diabetes	MST1	multiple	gene expression	[16]

The Hippo pathway was initially discovered in the fruit-fly *Drosophila melanogaster* by genetic screens for identifying genes required for growth and proliferation. The *warts* (*wts*) gene was first identified from the genetic mosaic screens, whose mutations caused dramatic overgrowth of the mutant tissues [17,18]. Subsequently, *salvador* (*sav*) and *hippo* (*hpo*) were discovered and, together with *wts*, defined a pathway controlling growth/proliferation and survival [19–24]. Hence, the Hippo pathway is also known as the Salvador–Warts–Hippo pathway.

Most components of the Hippo pathway are conserved from flies to mammals, although some differences may exist (details will be described below). Regulation of the Hippo pathway largely relies on kinase cascade of the core components. Inhibition of Hippo signalling (i.e. hypo-Hippo) means that the kinase cascade of the Hippo pathway is inactive, whereas hyperactivation of the Hippo pathway (i.e. hyper-Hippo) means that the kinase signalling cascade is active. The Hpo kinase (Ste20 family kinases, MST1/2 in vertebrates) forms a complex with the adaptor proteins Sav (SAV or WW45 in vertebrates) and Mats (Mob as tumour suppressor; MOB1A/B in vertebrates), which enhances Hpo/MST kinase activity and facilitates the interaction between Hpo/MST and the serine threonine kinase Wts (NDR family kinases, LATS1/2 in vertebrates). Hpo/MST phosphorylates and activates Wts/LATS via a sequential phosphorylation process [23,25–28]. Recent structural data have further proved the critical roles of MOB1 in this kinase activation loop through direct interactions with MST and LATS [26]. The activated Wts/LATS subsequently phosphorylates the final effector of the Hippo pathway, the transcriptional co-activator Yorkie (Yki; Yes associated protein (YAP) and TAZ in vertebrates) [29–37]. Phosphorylation of Yki/YAP/TAZ leads to cytoplasmic retention and subsequent protein degradation through β-TRCP (β-transducin repeat-containing E3 ubiquitin protein ligase)-dependent proteasomal degradation, thereby inhibiting their transcriptional activity [29,32,34,36,38–41]. Therefore, Yki/YAP/TAZ phosphorylation caused by active kinase cascade of the Hippo pathway indicates that Hippo signalling is hyperactivated. Recently, emerging evidence has uncovered additional kinases, which share similar roles with Hpo/MST and Wts/LATS in Hippo pathway regulation. MAP4K (mitogen-activated protein kinase kinase kinase kinase) kinases can activate LATS through a direct phosphorylation event [42,43]. Other members of the NDR family kinases, STK38 (NDR1) and STK38L (NDR2), also function as YAP kinases and inhibit YAP activity in certain cell types [44]. Similar to the LATS kinases, STK38 is activated by the binding of MOB1 as well as by MST-dependent phosphorylation [45–47]. Further studies will be needed to unravel the physiological roles of MAP4K and STK kinases in Hippo pathway regulation.

Phosphorylation-independent regulations also exist. Yki can directly bind to the FERM-domain-containing adaptor protein Expanded (Ex) and form a complex with Hpo/MST and Wts/LATS, thereby triggering the cytoplasmic retention of Yki [48,49]. Prior to these studies, Ex and Merlin (neurofibromin 2 (NF2) in vertebrates) were considered to function upstream of the core Hippo signalling cassette [50]. As an apical membrane-localized protein, Ex not only intrinsically modulates Yki activity but also transduces signal from outside of the cell through binding to the transmembrane protein Crumbs (Crb; CRB3 in vertebrates) [51–54]. The binding of Ex with Crb stabilizes its apical localization and then promotes the Hippo signalling pathway. Interestingly, however, vertebrate CRB3 regulates Hippo signalling through different mechanisms. CRB3 forms a complex with YAP/TAZ to sequester YAP/TAZ in the cytoplasm, thereby inhibiting their transcriptional activities. The prevention of YAP/TAZ nuclear localization by CRB3 may require components of junction-associated protein i.e. angiomotin family members (AMOT, AMOTL1 and AMOTL2), although the AMOT family has no homologue in *Drosophila*. AMOT proteins retain YAP/TAZ in the cytoplasm through direct (binding to YAP/TAZ) or indirect (stimulating LATS kinase activity) mechanisms [55–61]. Hence, in addition to kinase cascade regulation, the discovery of multiple protein–protein interactions indicates Hippo signalling is a regulatory network.

Conversely, when the Hippo pathway is inactivated, unphosphorylated Yki/YAP/TAZ translocates to the nucleus. Nuclear Yki/YAP/TAZ activates transcription through binding to Scalloped (Sd, the TEA domain family members 1–4 (TEAD1–4) in vertebrates) [62–66] or other transcription factors because Yki/YAP/TAZ lack their own DNA-binding domains. Binding to Sd/TEAD allows Yki/YAP/TAZ to activate expression of target genes that are involved in controlling cell growth, proliferation and survival [62–66]. Hence, the pathological role of the Hippo pathway in tumourigenesis is primarily caused by the aberrant activation of Yki/YAP/TAZ. On the contrary, little is known about how Yki/YAP/TAZ is inactivated in living cells (i.e. hyperactivation of the Hippo signalling pathway). In order to gain further understanding and consider potential disease implications of Hippo pathway activation, this review discusses the current knowledge of how and where the Hippo pathway is hyperactivated.

## 2. Hyperactivation of the Hippo pathway

Both intrinsic and extrinsic mechanisms modulate the Hippo pathway to maintain tissue homeostasis. This section will focus on recent advances in regulatory mechanisms that contribute to hyperactivation of the Hippo pathway.

### 2.1. Extracellular cues to regulate the Hippo pathway

The Hippo pathway can be regulated by various upstream stimuli, including G protein-coupled receptor (GPCR) signalling, adhesion cues through cell–cell contact, polarity, mechanical signals and cellular stress. Studies have built on observations that soluble hormones or growth factors act through GPCRs to activate or inactivate the Hippo pathway. Epinephrine, glucagon or dopamine receptor agonist can induce Hippo pathway activation through binding to Gαs and increase YAP phosphorylation [67]. However, in most cases, soluble factors have been shown to inhibit Hippo signalling activity. For instance, EGF and IGF can inhibit LATS and stimulate nuclear accumulation of YAP through phosphoinositide 3-kinase (PI3K) and pyruvate dehydrogenase kinase 1 (PDK1) signalling [68–70]. Lysophosphatidic acid (LPA), sphingosine 1-phosphate (S1P) and thrombin signal have been reported to act through G12/13 and Gq/11 to inactivate the Hippo pathway via stimulating Rho GTPases [67,71,72]. Moreover, Wnt ligands, such as Wnt5a/b and Wnt 3a, are found to mediate Gα12/13 and Rho to inhibit LATS activity, and then activate YAP/TAZ-dependent transcription. Many secreted Wnt inhibitors, including DKK1, BMP4, IGFBP4 and WNT5a/b, are YAP/TAZ target genes of this Wnt–Gα12/13-Rho–LATS–YAP/TAZ signalling (also called alternative Wnt-YAP/TAZ signalling) [73]. Therefore, alternative Wnt–YAP/TAZ signalling acts as a negative regulation of canonical Wnt signalling. Recently, studies have revealed several aspects regarding integrations between the Hippo pathway and Wnt pathway. As both signalling pathways are known to play essential roles in numerous cellular functions, understanding the crosstalk of Hippo and Wnt pathways may provide potential therapeutic targets.

Several upstream regulators established by cell–cell contact are known to promote the Hippo pathway by linking YAP to both adherens junctions (AJ) and tight junctions (TJ) proteins and reveal additional mechanisms of YAP inactivation. For example, homophilic binding of E-cadherin at AJ suppresses transcriptional activity of YAP by modulating MST activity [74]. An AJ component, α-catenin, has been shown to sequester YAP in the cytoplasm through physical interactions in mouse keratinocytes. Depletion of α-catenin leads to nuclear accumulation of YAP, thus triggering over-proliferative effects [75,76]. Recruitment of AMOT to AJ protein complexes (E-cadherin–catenin) also leads to cytoplasmic retention of YAP and stimulation of Hippo signalling, which is required to maintain pluripotent embryonic stem cells (ESCs) of inner cell mass in early blastocyst. Conversely, in outer cells of trophectoderm, the cell polarity restricts AMOT localization in the apical domain of outer cells, thereby inactivating the Hippo pathway [55]. In addition, fibronectin (an extracellular matrix protein) mediated cell–cell adhesion has been reported to act through focal adhesion kinase (FAK)–Src signalling to promote nuclear accumulation of YAP in a LATS-dependent manner. Inhibition of FAK–Src signalling is able to activate the Hippo pathway through cytoplasmic retention of YAP [77]. These studies indicate that differential inputs (e.g. adhesion cues or cell polarity) may regulate diverse cellular functions through modulating the Hippo pathway in distinct ways. The physical properties of cells, such as cell shape and cytoskeletal tension, have also been found to regulate the Hippo pathway [78,79]. GPCR-mediated regulation on YAP/TAZ activity is likely to act through modulating the actin cytoskeleton [67]. Disruption of the actin cytoskeleton activates Hippo signalling in a LATS-dependent manner [79–81]. By contrast, other studies proposed that this mechanical regulation controls YAP/TAZ activity independently of the LATS kinases, but through the Rho–Rock pathway [78,82–84]. This discrepancy could be due to different cell contexts or methodology, or the actual mechanism may involve LATS-dependent and -independent regulation of YAP/TAZ activity. Taken together, these findings illustrate how Hippo signal transduction is tightly regulated by the presence of neighbouring cells.

### 2.2. Intrinsic mechanisms for Hippo pathway activation

Intracellular mechanisms are also important for the activation of Hippo signalling. Although Wnt signal is initiated by secreted ligand binding, studies have revealed crosstalk between the Hippo pathway and Wnt pathway in various intracellular axes: (i) the cytoplasmic retention of phosphorylated YAP/TAZ interacts with Disheveled (DVL), thereby inhibiting Wnt target gene expression [85,86]; (ii) phosphorylated YAP/TAZ sequesters β-catenin in the cytoplasm [87], then β-TRCP is in turn recruited to this complex and triggers the proteasomal degradation of β-catenin [88,89]; and (iii) the tyrosine phosphatase SHP2 is restricted by phosphorylated YAP/TAZ in the cytoplasm of high density cells. Conversely, unphosphorylated YAP/TAZ facilitates nuclear translocation of β-catenin and SHP2, which in turn promotes Wnt/β-catenin target gene expression [90]. Therefore, phosphorylation of YAP/TAZ plays a critical role in controlling the integrated Hippo/Wnt signalling. Recent study has reported that protein kinase C zeta (PKCζ, belongs to atypical PKCs (aPKC)) can inhibit YAP and β-catenin via phosphorylation, indicating that PKCζ is a positive regulator to activate the Hippo pathway. This regulation is essential to maintain intestinal epithelial homeostasis [91]. Conversely, overexpression of aPKC stimulates Yki activity to promote cell proliferation and survival in *Drosophila* epithelial cells [52,54]. The discrepancy is also found in breast cancer cells where PKC acts downstream of an oestrogen-stimulated GPCR (G protein-coupled oestrogen receptor, GPER) to inhibit LATS, thereby activating YAP/TAZ [92]. In addition, different PKC isoforms can oppositely regulate the Hippo pathway in different cell types [93]. Conventional PKC (cPKC, including α, β and γ) inhibits LATS kinase activity while novel PKC (nPKC, including δ, θ, ε and η) promotes YAP/TAZ phosphorylation in HEK293A cells, HeLa cells and U251MG glioma cells. On the contrary, the effects of LATS-dependent YAP/TAZ phosphorylation in response to cPKC or nPKC are completely different in Swiss3T3 cells, MEF cells and A549 lung cancer cells [93]. It is likely that LATS-dependent phosphorylation is differentially regulated by different PKC isoforms in a cell type-specific manner.

The Hippo pathway has been shown to be regulated by the important transcriptional regulators of organogenesis, E proteins and ID proteins [94,95]. The widely expressed E proteins belong to the basic helix-loop-helix (bHLH) family, which heterodimerize with tissue-specific bHLH proteins to regulate cell growth, commitment and differentiation in many tissues [96,97]. The heterodimeric bHLH proteins bind to the E-box sequence (CANNTG) and drive gene expression. ID proteins lack a basic DNA-binding motif, so that the heterodimers of bHLH proteins with ID proteins inhibit their regulatory activities. Recent study in *Drosophila* has demonstrated that high levels of *Drosophila* E protein homologue (or loss of *Drosophila* ID protein) can activate *ex* transcription and promote Hippo signalling independent of any responses to cell–cell interactions. The hyperactivation of the Hippo pathway inhibits cell survival, which prevents progenitor cells from undergoing misspecified differentiation, thereby functioning as an intrinsic surveillance mechanism [94]. Previously, the binding with transcription factors (e.g. Sd/TEAD, Hth, RUNX, PAX, TBX5 and SMAD) is thought to be required for the transcriptional activity of Yki/YAP/TAZ and results in the inhibition of Hippo signalling [63–66,98–104]. Discovery of this regulation by which the Hippo pathway is hyperactivated by bHLH transcription factors may provide novel insights into the pathological involvement of hyperactive Hippo signalling.

In response to intracellular oxidative stress or DNA damage, nuclear translocated YAP binds to p73, a transcription factor that belongs to the tumour suppressor p53 family, and induces transactivation of proapoptotic genes such as PUMA and BAX, thereby triggering apoptosis [105–107]. A tumour suppressor, Ras association domain family 1A (RASSF1A), has been shown to mediate YAP phosphorylation and facilitate YAP–p73-mediated cell death [106]. The role of RASSF1A in YAP phosphorylation is thought to scaffold the interaction between MST and LATS and enables activation of their kinase activities [106,108]. In contrast with the common model that phosphorylated YAP is restricted in the cytoplasm, RASSF1A-dependent YAP phosphorylation induces nuclear translocation of YAP [106]. As decreased expression of RASSF1A has been reported in various cancers, which accounts for the tumour suppressor function of RASSF1A, these findings provide one possible mechanism by which elevated RASSF1A levels may contribute to apoptosis through enhancing MST–LATS–YAP phosphorylation (i.e. hyperactivation of the Hippo pathway). Future studies will be needed to address whether and how RASSF1A is induced in response to cellular stimuli. In addition, YAP can be phosphorylated by AKT (also known as protein kinase B), a downstream effector of PI3K signalling. Intriguingly, AKT-dependent YAP phosphorylation plays an opposing role in regulating YAP–p73 mediated apoptosis. AKT-dependent YAP phosphorylation results in a cytoplasmic retention of YAP, thereby attenuating YAP–p73 mediated apoptosis [105]. Collectively, these findings indicate that different sites of YAP phosphorylation may result in different subcellular localizations of phospho-YAP and cause distinct consequences of Hippo signalling [109], although the underlying mechanisms remain to be addressed in detail.

## 3. Prospective disease model of hyper-Hippo signalling

Over-proliferative effects caused by inactivation of Hippo signal transduction have been extensively studied in various cancers [1–6]. Conversely, the hyperactivation of the Hippo pathway is implicated in some human diseases. Cells with hyperactive Hippo signalling have been shown to undergo apoptosis and be eliminated *in vivo*. Excess cell death is often associated with neurodegenerative disease, ischaemia, autoimmune disease and metabolic disease [110]. This section includes current evidence, that has linked the hyperactivation of the Hippo pathway to human disease or disease models (table 1).

### 3.1. Hippo activation leads to adipogenesis in arrhythmogenic cardiomyopathy

Activation of the Hippo pathway has been linked to adipogenesis in the heart disease model. Increased levels of phosphorylated NF2, MST1/2, LATS1/2 and YAP have been detected in the myocardial samples with arrhythmogenic cardiomyopathy (AC) from human patients and mouse models. β-Catenin activity has been shown to be affected in AC, suggesting that the involvement of the Hippo pathway may also require the Wnt pathway [7]. AC, also known as arrhythmogenic right ventricular cardiomyopathy because it predominantly affects the right ventricular walls, is a hereditary cardiomyopathy that accounts for 15–25% of sudden cardiac deaths in patients younger than 35 years. The pathological hallmark of AC is the replacement of myocardium by fibroadipocytes, ventricular enlargement and dysfunction and lethal ventricular arrythmias. AC is a disease of the desmosomes, intercellular junctional complexes that join the ends of cardiomyocytes [111]. Desmosome disruption results in changes of mechanical control of upstream regulators of the Hippo pathway, leading to alteration of YAP/TAZ activity and localization [7]. Additionally, deregulated desmosome proteins have been considered to contribute to AC pathogenesis by enhancing adipogenesis driven by adipogenic transcription factor PPARγ [112]. The adipogenic effects can be antagonized by downregulation of Hippo kinases, indicating that hyperactive Hippo signalling could be a potential pathogenesis for AC [7]. This is consistent with the previous finding that adipogenesis can be induced by low YAP/TAZ activity [78,99]. It is likely that loss of mechanical integrity causes aberrant activation of the Hippo signalling pathway and promotes adipogenesis, thus resulting in AC. However, it is unclear whether desmosome mutation is exclusively required for Hippo-mediated AC pathology. Several questions remain to be addressed. For instance, is there Hippo pathway mutation(s) existing in AC patients? If so, is the Hippo pathway mutation(s) sufficient to trigger AC? It will be noteworthy to study in depth the underlying mechanisms in the future.

### 3.2. Degenerative disease

Sd/TEAD is the best-characterized DNA-binding partner of Yki/YAP/TAZ. The binding of Sd/TEAD with Yki/YAP/TAZ ensures its transcriptional activity [62–66]. Recent progress proves the interaction between YAP/TAZ and TEAD is essential for the maintenance and differentiation of retinal pigment epithelium in zebrafish [113]. Interestingly, a missense mutation in *TEAD1* has been identified that leads to Sveinsson's chorioretinal atrophy, a rare genetic disease in which choroid and retina are gradually degenerated [8,9]. This missense mutation disrupts the interaction of TEAD with YAP/TAZ and therefore blocks its transcriptional activity [9,62–66]. These findings imply that activation of the Hippo pathway (i.e. disruption of YAP/TAZ–TEAD function) may contribute to the pathogenesis of Sveinsson's chorioretinal atrophy and other ocular diseases. The Tondu-domain-containing proteins, *Drosophila* Vestigial (Vg) and Tgi (Vestigial-like proteins 1–4 (VGLL1–4) in vertebrates), also regulate Sd/TEAD-dependent transcription through physical interactions. Binding of Vg/VGLL1–3 with Sd/TEAD stimulates its transcriptional activity [114–117], whereas Tgi/VGLL4 acts as a repressor when interacting with Sd/TEAD and may compete for Yki/YAP/TAZ binding to Sd/TEAD [118,119]. This raises the possibility that disease-associated *TEAD* mutations might disrupt its binding to VGLL proteins, although this warrants further investigation.

In addition to the final effectors of Hippo signalling, Matsumoto *et al*. [4] demonstrate that MST2 kinase acts as a regulator to trigger photoreceptor apoptosis in a mouse model of retinal detachment [10]. Retinal detachment can cause permanent vision loss due to photoreceptor cell death. Apoptotic indications are reduced in *MST2* homozygous null mice after retinal detachment [10]. It will be interesting to further study whether (i) the effect of MST2 in retinal detachment requires YAP/TAZ activity and (ii) this regulation is involved in other retinal degenerative disorders.

Moreover, activation of MST1/2 has been connected to multiple neurodegenerative diseases. Alzheimer's disease (AD), the most common progressive neurodegenerative disease, is defined by the formation of amyloid plaques and neurofibrillary tangles in the brain and apoptotic cell death that causes synapse and neuron loss. AD pathogenesis is considered to be the accumulation and oligomerization of amyloid β (Aβ) peptide produced by defective proteolytic processing of the precursor of Aβ (AβPP) [120,121]. Recent study has reported that AβPP can promote nuclear translocation of a Forkhead transcription factor FOXO3a by inducing MST1-dependent phosphorylation of FOXO3a [11]. The nuclear FOXO proteins (e.g. FOXO1 and FOXO3) activate a pro-apoptotic member of Bcl-2 family and trigger an intrinsic apoptotic pathway, thus resulting in neuron death [11,122]. The MST–FOXO-mediated neuron death could be considered as a branch of the Hippo pathway. Activation of MST kinase is also induced by oxidative stress, which has been associated with various diseases including neurodegeneration [122–124]. Knockdown of FOXO can rescue MST1 overexpression- or oxidative stress-induced neuron death [125], supporting that FOXO acts as a downstream effector of MST1. However, it remains unclear whether depletion of MST1 is sufficient to rescue the AβPP-mediated neuron death. AKT has also been reported to phosphorylate FOXO, whereas AKT-dependent FOXO phosphorylation blocks the kinase activity of MST1 towards FOXO [126]. In contrast with MST1 phosphorylation, FOXO phosphorylation by AKT promotes its cytoplasmic retention, thereby preventing FOXO-mediated apoptosis [127–131]. In addition, activation of AKT can prevent the toxic effect of AβPP [132–134]. Collectively, these studies suggest a protective role of AKT in AβPP-mediated neuron death. Notably, AKT activation is inhibited by MST phosphorylation, indicating a mutual inhibition between MST and AKT kinases in FOXO regulation [126]. Intriguingly, YAP/TAZ has been proposed to act as downstream mediators of amyloid precursor protein signalling through physically interacting with the amyloid precursor protein and forming a transcriptionally active protein complex [12]. These findings may link the core components of the Hippo pathway to AD pathogenesis, although the detailed mechanisms await further investigation.

MST1 kinase has also been reported as an important regulator in skeletal muscle atrophy caused by denervation, ageing and metabolic diseases. Upregulation of MST1 induces muscle atrophy through phosphorylating and inducing nuclear accumulation of FOXO3a, thereby activating multiple autophagy genes. Furthermore, the neurogenic atrophy can be attenuated in *Mst1* homozygous null mice [13]. Amyotrophic lateral sclerosis (ALS) is a severe progressive neurodegenerative disorder that involves the death of motor neurons. When the motor neurons die, patients progressively lose the ability to control muscle movement, thereby disrupting speech, eating, moving and breathing. The elevation of MST1 activity has been reported in motor neurons from a mouse model of ALS [14]. Mutations of Cu/Zn superoxide dismutase type-1 (SOD1), a crucial enzyme for cellular antioxidant defence mechanisms, have been linked to a hereditary form of ALS [135]. ALS-associated *SOD1* (G93A) mutant induces MST1 activation in neurons in an oxidative stress-dependent manner. Increased MST1 causes autophagosome accumulation and death of motor neurons through activation of p38 and caspases. ALS phenotypes can be attenuated when MST1 is depleted in an ALS mouse model [14], indicating that MST1 activity plays a critical role for ALS pathogenesis. In this regard, future studies on the pathogenic roles of MST1 in ALS will inspire a potential route for targeting therapies. Moreover, YAP–p73-mediated neuron death has been reported in an ALS mouse model [15]. These findings indicate that the pro-apoptotic signalling mediated by MST1–YAP–p73 not only causes multiple types of neurodegenerative disorders but also suppresses tumourigenesis in certain tumour types [106,107,136,137].

### 3.3. Metabolic disorder

Recent study has revealed that hyperactivation of MST1 kinase plays an essential role in triggering the initiation of β-cell death and disruption of insulin secretion, thereby resulting in diabetes [16]. The destruction of insulin-producing β-cells caused by apoptotic cell death is a hallmark of both type 1 and type 2 diabetes. In diabetic human and mouse β-cells, MST1 is highly activated and directly phosphorylates the critical β-cell transcription factor, pancreatic and duodenal homeobox 1 (PDX1), leading to the subsequent degradation of PDX1 and insulin secretion failure. Notably, loss of MST1 is sufficient to rescue survival and insulin tolerance of pancreatic β-cells through preservation of PDX1 [16]. *Mst1^−/−^* mice are also protected from diabetogenic stimulation, suggesting MST1 could be a potential therapeutic target for treating diabetes. TEAD–YAP has been shown to activate key pancreatic transcription factors such as PDX1 [138]. However, it is currently unknown whether YAP/TAZ activity is inhibited in the MST1-mediated β-cell death. Hence, it will be attractive to elucidate the cellular mechanisms by which MST1 is hyperactivated under diabetic stimuli.

### 3.4. Infertility disease

Polycystic ovarian syndrome (PCOS) is the most common endocrine disorder among reproductive-age women, which results in infertility, menstrual disorders, metabolic symptoms and endometrial cancer. Although PCOS has been strongly suggested as a genetic disease, the pathology and cause of PCOS is largely uncertain. In patients with PCOS or primary ovarian insufficiency (POI), infertility treatment has been proposed to promote ovarian follicle growth. Facilitating ovarian follicle growth by promoting actin polymerization is sufficient to induce the nuclear translocation of YAP and subsequent activation of the downstream target genes BIRC (baculoviral inhibitors of apoptosis repeat containing) and CTGF (connective tissue growth factor) in a PCOS mouse model and POI patients [139,140]. Hence, inhibition of Hippo signal transduction provides a treatment for ovarian disorders, although the underlying mechanism remains elusive. It will be noteworthy to further address: (i) whether the Hippo pathway is hyperactivated in defective ovaries, (ii) which component(s) of the Hippo pathway is deregulated in animal models or human patients with defective ovaries and (iii) whether the pathogenesis of PCOS or POI is caused by decreased cellular proliferation and/or increased apoptosis upon Hippo pathway activation.

### 3.5. Disease implications of helix-loop-helix proteins and the Hippo pathway

Deregulated E proteins and ID proteins are known to contribute to a variety of diseases [95,141–143]. Therefore, the discovery of the regulatory mechanism between E/ID proteins and the Hippo pathway [94] pinpoints an important insight for the physiological control to maintain organ integrity. To date, there has been no direct evidence linking the hyperactivated Hippo pathway, HLH proteins and disease, but there are some intriguing clues for the potential involvement in diseases. For instance, *Id4* homozygous null (*Id4^−/−^*) mice have been shown to enhance adipogenesis and reduce osteogenenic differentiation [144]. Similarly, depletion of *TAZ* has been reported to promote adipogenesis [99]. The promising phenotypes may motivate further investigations to study whether the aforementioned intrinsic regulation involving the HLH proteins–Hippo pathway axis can promote adipogenesis. Future studies may also extend the current view that the involvement of YAP/TAZ in mesenchymal stem cell differentiation (adipogenesis and osteogenesis) is governed by mechanical cues.

A role for ID proteins has been suggested in circadian rhythm. ID proteins have been reported to regulate circadian rhythm through sequestering the circadian bHLH transcription factors CLOCK and BMAL, thus reducing expression of the *Period* (*Per*) clock protein [145,146]. By modulating the activity of CLOCK, BMAL and Per, casein kinase 1 (CK1) has been thought to be an important regulator for circadian rhythms. Intriguingly, CK1 promotes YAP/TAZ degradation through β-TRCP E3 ligase [38,41], suggesting YAP/TAZ may be implicated in circadian regulation. Deregulation of β-TRCP E3 ligase-dependent protein degradation has been reported to contribute to autosomal dominant polycystic kidney disease (PKD), which is frequently caused by inactivating mutations in the *PKD1* and *PKD2* genes [147]. *TAZ* homozygous null mutant mice display symptoms of PKD due to accumulation of product of the *PKD2* gene, polycystin-2. TAZ binds and targets polycystin-2 for degradation through the β-TRCP E3 ligase pathway [148]. Polycystin-2 can sequester ID proteins in the cytoplasm through direct interaction. PKD phenotypes have been observed when the interaction between polycystin-2 and ID2 is disrupted [149]. Taken together, these findings suggest that the crosstalk between E/ID proteins and the Hippo pathway may be involved in circadian rhythm and PKD. However, the connections between these lines of evidence are only correlations without clear mechanistic support. Further studies are needed to characterize the connection and involvement of YAP/TAZ and ID proteins in circadian cycles and PKD pathogenesis.

**Figure 2.**
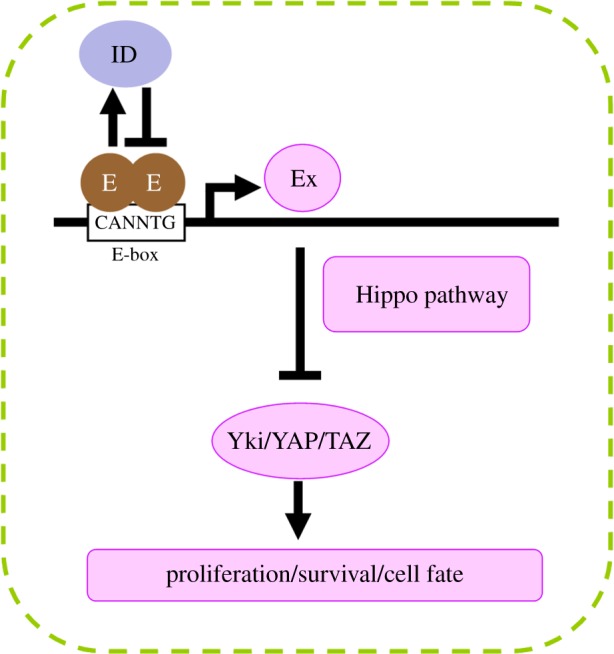
Intrinsic Hippo activation by *Drosophila* HLH proteins. Depletion of *Drosophila* ID protein results in elevated *Drosophila* E protein. The high levels of E protein activate *ex* transcription through binding to the E-box sites in the cis-regulatory element, thereby activating the Hippo pathway. The hyperactivated Hippo pathway prevents cellular proliferation and survival, leading to the elimination of misspecified progenitor cells.

*Id3* homozygous null mice have been reported to induce features of Sjogren's syndrome [150], a chronic autoimmune disease, which disrupts salivary and lachrymal glands. Unexpectedly, the nuclear accumulation of TAZ has been reported to be sufficient to cause Sjogren's syndrome [151]. Although this is in opposition to the current knowledge of the HLH–Hippo regulatory aspect (figure 2), it remains interesting to determine the potential connection between E/ID proteins and Hippo pathway in Sjogren's syndrome. For instance, it will be useful to understand whether depletion of *TAZ* in *Id3^−/−^* mice relieves the symptoms of Sjogren's syndrome.

## 4. Conclusion

At present, most dysfunctions of upstream regulators, kinases and downstream effectors of the Hippo pathway often lead to inhibition of Hippo signal transduction and are associated with overproliferative disorders such as cancer. However, aspects regarding activation of the Hippo pathway are also fundamental. By describing the recent advances in disease implications of Hippo activation, this review attempts to inspire future research of the underlying mechanisms and potential therapeutics for these diseases. Accumulating evidence has strongly linked *TEAD1* disruption or MST kinases activation to many diseases, such as Sveinsson's chorioretinal atrophy, AC, AD, skeletal muscle atrophy, ALS and diabetes. Nevertheless, how these diseases are related to the roles of the Hippo pathway in normal development is still unclear and remains to be investigated. Small molecule inhibitors targeting Hippo pathway components have been reported to suppress tumourigenesis in various cancer cell lines [1]. However, some molecules may function oppositely. For instance, compound 9E1 has been shown to inhibit MST1 kinase activity and LPA, S1P or thrombin can target GPCR signalling to inhibit LATS kinase activity, thus promoting transcriptional activity of YAP/TAZ [67,71,72,152]. Therefore, these kinase inhibitors could be considered as potential therapeutic strategies to treat disorders caused by hyperactive Hippo signalling.

Interestingly, aberrant activation of MST kinases appears to cause a broad range of diseases occurring in multiple organs. For instance, MST activation can result in AD and diabetes. A possible mechanistic link between diabetes and AD is suggested by studies indicating that type 2 diabetes patients have a higher risk of developing AD [153]. It is interesting to speculate that hyperactive Hippo signalling is a common cause of diverse diseases in different organs. Future studies will be required to determine whether and how the Hippo pathway becomes globally hyperactive *in vivo*.

Apoptosis caused by hyperactive MST has been shown to act through FOXO (FOXO3) or AKT in some cases, although it remains to be tested if, and how, YAP/TAZ is involved in MST–FOXO- or MST–AKT-mediated apoptotic signalling. Interestingly, it has been reported that YAP can act as a transcriptional co-activator of FOXO1 in regulating the transcription of antioxidant genes in cardiomyocytes. Thus, hyperactive Hippo signalling stimulates cell death by inhibiting YAP–FOXO1-mediated gene expression in response to oxidative stress [154]. Recent work illustrates that dysregulated HLH transcription factors are sufficient to activate the Hippo signalling pathway. Results from this work demonstrate that the hyperactive Hippo pathway indeed contributes to apoptosis dependent upon the elimination of Yki/YAP/TAZ activity [94]. So far, connections between HLH proteins and core components of the Hippo pathway have not been proposed as direct causes of particular diseases or defects. However, independent evidence has implicated the roles of HLH proteins or Hippo components in adipogenesis, circadian regulation and Sjogren's syndrome. It would be informative to substantiate the association of HLH proteins with Hippo components in adipogenesis, circadian regulation, Sjogren's syndrome and other potential diseases. Clear challenges remain to clarify the pathogenesis at the molecular and cellular level.
